# Antimicrobial resistance due to the content of potentially toxic metals in soil and fertilizing products

**DOI:** 10.1080/16512235.2018.1548248

**Published:** 2018-12-11

**Authors:** Siamak Yazdankhah, Eystein Skjerve, Yngvild Wasteson

**Affiliations:** a Norwegian Institute of Public Health (NIPH), Norwegian Scientific Committee for Food and Environment, Oslo, Norway; b Faculty of Veterinary Medicine, Department of Food Safety and Infection Biology, Norwegian University of Life Sciences, Oslo, Norway

**Keywords:** Potentially toxic metals, antimicrobial resistance, fertilizing products, manure, sewage, environment

## Abstract

Potentially toxic metals (PTM), along with PTM-resistant bacteria and PTM-resistance genes, may be introduced into soil and water through sewage systems, direct excretion, land application of biosolids (organic matter recycled from sewage, especially for use in agriculture) or animal manures as fertilizers, and irrigation with wastewater or treated effluents. In this review article, we have evaluated whether the content of arsenic (As), cadmium (Cd), chromium (CrIII + CrVI), copper (Cu), lead (Pb), mercury (Hg), nickel (Ni), and zinc (Zn) in soil and fertilizing products play a role in the development, spreading, and persistence of bacterial resistance to these elements, as well as cross- or co-resistance to antimicrobial agents. Several of the articles included in this review reported the development of resistance against PTM in both sewage and manure. Although PTM like As, Hg, Co, Cd, Pb, and Ni may be present in the fertilizing products, the concentration may be low since they occur due to pollution. In contrast, trace metals like Cu and Zn are actively added to animal feed in many countries. In several studies, several different bacterial species were shown to have a reduced susceptibility towards several PTM, simultaneously. However, neither the source of resistant bacteria nor the minimum co-selective concentration (MCC) for resistance induction are known. Co- or cross-resistance against *highly important antimicrobials* and *critically important antimicrobials* were identified in some of the bacterial isolates. This suggest that there is a genetic linkage or direct genetic causality between genetic determinants to these widely divergent antimicrobials, and metal resistance. Data regarding the routes and frequencies of transmission of AMR from bacteria of environmental origin to bacteria of animal and human origin were sparse. Due to the lack of such data, it is difficult to estimate the probability of development, transmission, and persistence of PTM resistance.

**Abbreviations:** PTM: potentially toxic metals; AMR: antimicrobial resistance; ARG: antimicrobial resistance gene; MCC: minimum co-selective concentration; MDR: multidrug resistance; ARB: antimicrobial resistant bacteria; HGT: horizontal gene transfer; MIC: minimum inhibitory concentration

## Introduction

In the last decade, we have witnessed a dramatic increase in both the proportion and absolute number of bacterial pathogens presenting multidrug resistance (MDR) to antimicrobial agents. Organizations such as the US Centers for Disease Control and Prevention (CDC), the European Centre for Disease Prevention and Control (ECDC), and the World Health Organization (WHO) consider those infections caused by multidrug-resistant bacteria as threatening global disease and major public health concerns [1].

In environmental ecosystems, potentially toxic metals (PTM)/heavy metal contaminants may interact with native microorganisms residing in the same ecosystems. These organisms have developed resistance mechanisms that allow them to survive and, in some instances, to remove/reduce the contents of contaminants. The co-occurrence of antimicrobial resistance (AMR) and metal resistance in bacteria have been reported in many review articles [2–6]. This co-occurrence is caused by cross- and co-resistance phenomena. Cross-resistance occurs when the same mechanism simultaneously reduces the susceptibility to metals and antimicrobial agents used in therapy, and co-resistance occurs when separate resistance genes are situated on the same genetic element [2]. Some studies suggest that metal contamination in natural environments could have an important role in the maintenance and proliferation of AMR [7,8]. This is of particular concern, considering that PTM/heavy metals of anthropogenic origin, such as agricultural and aquacultural practices, are currently found at several orders of magnitude greater than levels of pharmaceutically produced antimicrobials [9]. Unlike pharmaceutically produced antimicrobial agents, metals are not subject to degradation and therefore represent a long-term selection pressure. Thus, there are concerns regarding the potential of metal contamination to maintain a pool of AMR genes in both natural and clinical settings. After use, antimicrobials, including PTM, along with antimicrobial-resistant bacteria (ARB) and antimicrobial resistance genes (ARGs), including genes encoding resistance against heavy metals, may enter soil and water through sewage systems, direct excretion, land application of biosolids (organic matter recycled from sewage, especially for use in agriculture) or animal manures as fertilizers, and irrigation with wastewater or treated effluents. The presence of active antimicrobial compounds and their metabolites and toxic/heavy metals in environmental compartments may also select for resistance in environmental bacterial communities or microbiota.

All use of antimicrobials, including biocides and toxic metals, in human and veterinary medicine, including aquaculture and agriculture, may be drivers for the development and dissemination of AMR in bacteria. Whereas AMR properties in bacteria are transferred from one generation to the next by vertical gene transfer within the same bacterial species, horizontal gene transfer (HGT) of AMR may occur both within the same species and between different bacterial species, including unrelated bacterial species. HGT may occur within and between bacterial species by conjugation, transformation, or transduction, as has been described extensively in review articles [10,11,12].

This review article is based upon a report requested by the Norwegian Food Safety Authority and assessed by Norwegian Scientific Committee for Food and Environment. The report is available on www.vkm.no. The aim of this article is to review whether the content of arsenic (As), cadmium (Cd), chromium (CrIII + CrVI), copper (Cu), lead (Pb), mercury (Hg), nickel (Ni), and zinc (Zn) in soil and fertilizing products play a role in the development, spreading, and persistence of bacterial resistance to these elements, as well as cross- or co-resistance to antimicrobial agents.

## Methods; literature search strategy

For review articles: the search was conducted in PubMed using the terms: heavy metals AND antimicrobial resistance AND review, using the Advanced Search Builder provided in PubMed (www.ncbi.nlm.nih.gov/pubmed), limited to the period 1999–2017. This resulted in 156 hits (20. January 2017). For original articles: the search was conducted in PubMed using the terms: different heavy metals listed in Table 1 [Title/Abstract] AND antimicrobial resistance AND sewage or manure using PubMed. This resulted in 89 (sewage = 65, manure = 24) hits for all PTM assessed in this report (20. January 2017).

**Table 1.** Mechanisms of action of potentially toxic metals in bacteria.

Toxic metal	Mechanisms of action
Arsenic*, **	As is a toxic metalloid that exists in two major inorganic forms: arsenate and arsenite. Arsenite disrupts enzymatic functions in cells, while arsenate behaves as a phosphate analogue and interferes with phosphate uptake and utilization [64].
Cadmium**	Cd is the most toxic heavy metal, especially against microorganisms. The effects may be summed up under the general headings: “thiol-binding and protein denaturation”, “interaction with calcium metabolism and membrane damage”, “interaction with zinc metabolism”, and “loss of protective function”. The *dsbA* encoding gene for a product required for disulphite formation, leads to Cd resistance in Gram-negative bacteria [4].
Chromium***	Cr is a micronutrient metal and may be toxic when its concentration exceeds requirements. As a transition metal, it exists in different valency states, ranging from – II to +VI, with Cr(VI) and Cr(III) being the dominant species in the environment. Out of two commonly occurring states, Cr(VI) is toxic to biological systems due to its strong oxidizing potential that invariably damages the cells [65]. Cr(VI) is known to be harmful to all forms of living systems [66], including microorganisms [67].
Copper***	Cu interacts readily with molecular oxygen. Its radical character makes Cu very toxic. Cu toxicity is based on production of hyperoxide radicals and on interaction with cell membranes [4].
Lead	Pb has a low biological available concentration due to its low solubility. Thus, Pb is not extraordinarily toxic to microorganisms [4]. Some forms of lead-salt, like lead acetate or nitrate, induce mutagenicity and DNA breaks at a toxic dose in some bacterial species [68].
Mercury**	Hg toxicity has been attributed to the inactivation of enzymes and interference with other protein functions by the tight binding of mercuric ions to thiol and imino nitrogen groups in these, or the displacement of other metal cofactors from enzymes. Mercuric ions also bind to nucleotides and lipids, interfering with DNA function and contributing to lipid peroxidation. Mercuric ions and organomercurials have the ability to pass rapidly through biological membranes, and organomercurials are highly lipid soluble [69].
Nickel	Four mechanisms of Ni toxicity have been proposed: 1) Ni replaces the essential metal of metalloproteins; 2) Ni binds to catalytic residues of non-metalloenzymes; 3) Ni binds outside the catalytic site of an enzyme to inhibit allosterically; and 4) Ni indirectly causes oxidative stress [70]. Oxidative stress to Ni toxicity in microorganisms is known and some studies have shown that cells subjected to oxidative stress exhibit enhanced DNA damage, protein impairment, and lipid peroxidation, along with increased titres of oxidative stress defence systems; reviewed by [71].
Zinc**	Zn ions inhibit multiple activities in bacterial cells, such as glycolysis, transmembrane proton translocation, and acid tolerance [72]. Trace elements like Zn may be toxic to bacteria and this may be due to their chemical affinity to thiol groups of macro-biomolecules, but may also be dependent on the solubility of the metal compounds under physiological conditions; reviewed by [6].

* Arsenic is not a true metal, but a semi-metal (a semi-metal or metalloid is a chemical element that has the properties of both metallic and non-metallic elements) ** As, Hg, Cd are considered non-essential elements in living organisms. *** Cu, Zn, and Cr are also essential metals in living organisms.

For further search, the terms used were: different potentially toxic metals/heavy metals AND antibiotic resistance or antibiotic resistant AND organic fertiliser/fertilizer or ‘waste’ or ‘effluent’. Only 12 citations that had not previously been identified and fulfilled the criteria for inclusion were obtained. In addition to the articles obtained by the primary searches, a few other relevant articles were found referenced in the initially identified articles and were also included.

For review articles, we limited our search to the period 1999–2017. For original articles, due to limited numbers of articles identified, all articles from the search strategy were included. Articles describing development of resistance in microorganisms other than bacteria, such as viruses, fungi, and parasites were excluded. Articles that were not in English or a Scandinavian language (Swedish, Danish, and Norwegian) were also excluded. The titles and abstracts of all literature sources identified were screened by one person and those that did not relate to the scope of this article were excluded. For articles of potential relevance, the full text was obtained and assessed for relevance to this overview.

Review articles that focused on bacteria with reduced susceptibility against As, Cd, Cr, Cu, Ni, Hg, Pb, or Zn were included. These review articles were used mainly to present information regarding mode of action and mechanisms of resistance. When using the search terms ‘antimicrobial resistance’ AND ‘heavy metals’ AND ‘fertilizers’, no articles were identified. Therefore, we used the search terms ‘manure’ or ‘sewage sludge’, which include organic fertilizers.

## The review

### Terminology

Antimicrobial susceptibility testing with phenotypic methods is based on the measurement of the minimum inhibitory concentration (MIC) with the use of defined clinical breakpoints to categorize the test organism as susceptible, intermediate, or resistant. Phenotypic antimicrobial susceptibility testing requires an agreement on breakpoints and a rigorous standardization of methods and materials [13]. Standardization of methods and materials for antimicrobial agents used in therapy and prophylaxis is performed by the European Committee for Antimicrobial Susceptibility Testing, EUCAST (http://www.eucast.org) in Europe, and by Clinical Laboratory Standard Institution, CLSI (http://clsi.org/m100/) in USA. The standardization includes many experimental parameters, such as preparation of media, inocula, inoculation of agar plates, application of antimicrobial discs, incubation of plates, examination of plates after incubation, measurement of inhibition zone diameters, and interpretation of results, and quality controls [14]. Such standardized methods for determination of MIC-values for PTM have still not been established, although official methods for the determination of toxic metals concentration in feed and food exist (https://ec.europa.eu/jrc/en/eurl/heavy-metals/legislation). Some limitations regarding determination of toxicity of toxic metals to bacteria have been discussed elsewhere [15].

Our knowledge regarding the activity of PTM against the different bacterial species present in fertilizing products and in soil is limited. Data regarding the biological effects of sub-inhibitory concentration of toxic metals, which for some antimicrobials are known to induce resistance in different bacterial species at the laboratory level, have not been identified in publications included in this article.

Terms such as ‘resistance’ and ‘tolerance’ have acquired specific technical meanings in the field of antimicrobials. The current terminology in microbiology distinguishes between clinical and microbiological antimicrobial resistance, particularly for antimicrobials used for therapy and/or prophylaxis. Clinical resistance is present when phenotypic testing of a microbe/antimicrobial combination against a clinical breakpoint indicates that therapeutic failure is likely, even with maximal dosing. Microbiological resistance is defined by the presence of an acquired or mutational resistance mechanism to the drug in question, in comparison with a fully susceptible ‘wild-type’, and may be assessed by genetic analysis or phenotypic testing against a wild-type cut-off value. The clinical resistance scenario is clearly not applicable in the case of PTM, so, to avoid ambiguity, it is desirable to avoid using ‘resistance’ in relation to the activity of these agents. However, in this document we use the term ‘resistance’ since other terms have not yet been established, yet. Similarly, the non-specific use of the term ‘tolerance’ is discouraged. The preferred terminology of many authors concerning variation in the effects of PTM upon bacteria is ‘reduced/increased susceptibility’, or variants thereof [16].

In 2012, Seiler and Berendonk introduced the minimum co-selective concentration (MCC) of a metal [5], with MCC defined as the minimum toxic metal concentration that correlates with detection of increased bacterial antibiotic resistance in co-regulation of a bacterial community/environment.

Most data regarding other antimicrobial agents, like biocides and toxic metals are obtained from studies using planktonic phase microorganisms rather than microorganisms in more natural conditions such as in a biofilm. Notably, gene expression in microorganisms living in a biofilm differs from that in planktonic cells, and the concentration of a compound needed to kill microorganisms in biofilms may be 10–500 times higher than in the planktonic phase [17].

### Heavy metals OR potentially toxic metals

Metals can be classified into four major groups based on their health importance [18]:

- Essential: Cu, Zn, Co, Cr, Manganese (Mn), and Iron (Fe). These metals are also called micronutrients and are toxic in organisms when taken in excess of requirements.
- Non-essential: Barium (Ba), Aluminium (Al), and Lithium (Li)
- Less toxic: Tin (Sn) and Al
- Highly toxic: Hg, Cd, As. As is a metalloid with both metallic and non-metallic properties, but will be included under the PTM group in this document.

Some metals have been used as antimicrobial agents since antiquity, but their modes of action differ from those of classical antimicrobial agents. Among these metals, Zn, Ni, Cu, and Cr are toxic metals with high to moderate importance as trace elements, while As, Cd, Hg, Pb have no beneficial functions in this context and should be considered entirely as toxic [4].

According to the International Union of Pure and Applied Chemistry, the term ‘heavy metal’ is a ‘meaningless term’ because there is no standardized definition of a heavy metal [19]. Some light metals or metalloids are toxic, but some high-density metals are not. For example, cadmium is generally considered a heavy metal, with an atomic number of 48 and specific gravity of 8.65, whereas gold is typically not toxic, but has an atomic number of 79 and a specific gravity of 18.88. For any given metal, the toxicity varies widely, depending on the allotrope or oxidation state of the metal. Most heavy metals are naturally occurring elements, usually with high atomic weight and a density at least 5 times greater than that of water. Because of confusion regarding the term ‘heavy metals’, we use the term ‘potentially toxic metals’ (PTM) rather than heavy metals throughout this article.

### Organic and inorganic fertilizers

A ‘fertilizing product’ is a substance, mixture, microorganism, or any other material, applied or intended to be applied, either on its own or mixed with another material, on plants or their rhizosphere for the purpose of providing plants with nutrients or improving their nutritional efficiency. Commercial phosphate (P) fertilizers and ‘agricultural liming materials’ contain low concentrations of PTM contaminants. Animal manures and sewage sludge (biosolids), both treated and untreated, are the main organic fertilizers that may contain PTM contaminants, whereas inorganic fertilizers mainly contain Cd. PTM in biosolids may be found in the inorganic form or may be organically complexed, which could affect their toxicological profile and stability, and their chemical reactions in soil. These PTM may accumulate in soil with repeated fertilizer applications [20].

### Mode of action of toxic metals

In a metal, atoms readily lose electrons to form cations that are surrounded by delocalized electrons. This behaviour is responsible for the conductivity and antimicrobial effects of metals [21]. Metals may be toxic to bacteria, and this microbial toxicity may be due to their chemical affinity for thiol groups of macro-biomolecules, but also depends on the solubility of the metal compounds under physiological conditions [22]. Several possible modes of action of toxic metals have been reported, including: protein dysfunction; production of reactive oxygen species (ROS) and antioxidant depletion; impaired membrane function; interference with nutrient uptake and genotoxicity. Mechanisms of action related to specific metals are described in Table 1.

### Mechanisms of resistance

In order to avoid cellular toxicity from elevated exposure to potentially toxic metals, bacteria have evolved mechanisms of metal resistance. The mechanisms of resistance to toxic metals are presented in detail in the review article of Seiler and Berendonk [5]. The authors concluded that, like antimicrobial agents, toxic metals might promote the spread of AMR via co-selection.

Resistance mechanisms for PTM may be divided into the following three groups:

1. **Complex formation** or sequestration of toxic metals [23]. By selective binding with macromolecules, the concentration of the free toxic ions in the cytoplasm is reduced. Biosorption of toxic metals is known from cell membranes, cell walls, and extracellular polymeric substance (EPS) of biofilms [24]. For example, the EPS matrix and the polysaccharides contained in biofilm have been reported to bind toxic metals [25]. Thus, the metal tolerance of bacteria belonging to that biofilm is enhanced.
2. **Detoxification** through reduction of intracellular ions [4]. A well-understood example is mercury reductase, encoded by the *merA* gene. The MerA protein reduces Hg^2+^ ions to the less toxic Hg^0^ [26]. Hg^0^ will then diffuse out of the cell, due to its low evaporation point [4].
3. **Excretion of toxic ions** by efflux systems [27]. The cation/proton antiporter Czc, known, for example from *Alcaligenes eutrophus*, mediates resistance to the metal ions Cd^2+^, Zn^2+^, and Co^2+^ by removal of metals from the cytoplasm though the inner and outer membranes to the surrounding environment [23].

These mechanisms are considered in greater detail for the PTMs assessed in this article in Table 2.

**Table 2.** Mechanisms of resistance against different toxic metals assessed in this report.

Toxic metal	Mechanisms of resistance
Arsenic	As tolerance in bacteria is usually mediated by the gene products of the widespread extensively studied *ars* operon [73,74]. Although the organization of the *ars* operons varies greatly between strains, there are some core genes that are almost always present: the simple gene set conferring basal resistance consists of the three-gene operon *arsRBC* as present in the *E. coli* genome [73] and on *S. aureus* plasmid pI258 [75]. Mechanisms of resistance against As in bacterial species have been reviewed by [76] and [33]. The main cross-resistance between As and antimicrobial agents may be activation of efflux pumps.
Cadmium	Resistance against Cd in bacteria is based on Cd efflux. In Gram-negative bacteria, Cd seems to be detoxified by an RND-driven system like Czc, which is mainly a Zn exporter, and Ncc, which is mainly a Ni exporter. Resistance against Cd in *S. aureus* and other Gram-positive bacteria is associated with a CdA pump or other CdA-like proteins [4].
Chromium	Both prokaryotic and eukaryotic microorganisms respond to Cr(VI) challenge by combining cellular networks acting at several levels, such as the reducing power generated by basal energy metabolism, iron and sulphur acquisition and homeostasis, protein oxidative stress protection, DNA repair, efflux pumps like *chrA*-encoding efflux pump orthologues, detoxification enzymes [77].
Copper	Resistance to Cu has been reported, both in bacteria isolated from humans and animals, and in bacteria of environmental origin. Resistance against Cu may be linked to resistance against several antimicrobials, for example macrolides including erythromycin (*erm*) [78–80] or glycopeptides such as vancomycin (van) [81] in enterococci. Resistance towards Cu is frequently encoded by genes located on plasmids and transposons, and is often transferable between bacterial species. Such resistance genes may be transferred to other bacteria and co-selection may occur.
Lead	To diminish its high toxicity, microorganisms have developed several mechanisms that allow them to survive exposure to Pb(II). The main mechanisms of Pb resistance involve adsorption by extracellular polysaccharides, cell exclusion, sequestration as insoluble phosphates, and ion efflux to the cell exterior [82,83].
Mercury	In Gram-negative enteric bacterial species, Hg-resistance genes are often found on plasmids and are associated with transposons/integrons [84–86]. Similar mobile units are found in *S. aureus* and enterococci [84,87]. More recently, oral streptococci and other oral genera have been shown to have reduced susceptibility to Hg, although, in general, the mechanisms of resistance have not been identified [8].
Nickel	Ni efflux pumps are best characterized in organisms exhibiting hyper-resistance to this metal, typically isolated from soils. Two examples of Ni-resistant organisms obtained from metal-contaminated industrial sites are *Cupriavidus* (formerly *Wautersia, Ralstonia*, or *Alcaligenes*) *metallidurans* and *Alcaligenes* (or *Achromobacter*) *xylosoxidans*. Ni-efflux pumps also are present in non-extremophiles, as exemplified by *E. coli* and *H. pylori*. Although Ni efflux is widely used by cells to protect against elevated concentrations of this metal, several other mechanisms are utilized by various microorganisms, and have been reviewed by Macomber and Hausinger [70].
Zinc	Resistance against Zn has been reported, both in Gram-positive bacteria like MRSA [63] and Gram-negative bacteria like *E. coli* [88]. Resistance to Zn, which is mainly associated with the *czrC* gene, has been reported in bacteria isolated from humans, animals, and from the environment. Resistance against Zn may be linked to resistance against methicillin in *S. aureus* (Cavaco et al., 2011) and Zn supplementation in animal feed may increase the proportion of multi-resistant *E. coli* in gut microbiota [88].

A database of antibacterial biocide- and metal-resistance genes has been established, based on an in-depth review of the scientific literature, by Pal et al [28]. The BacMet database (http://bacmet.biomedicine.gu.se) contains several hundred experimentally verified resistance genes [28]. In addition, the database also contains more than 25,000 potential resistance genes obtained from public sequence repositories. All resistance genes in the BacMet database have been organized according to their molecular function and induced resistance phenotype. This collection of genes enables correlations between metal resistance and antimicrobial resistance to be made, by investigating how often old and new emerging strains carrying either one of both type of resistances simultaneously.

Many of the PTM assessed in this article are among the metals with the highest number of known resistance genes reported by Pal et al. [28]. The BacMet database may facilitate research to improve our understanding of co- and cross-resistance of biocides and metals to antibiotics within bacterial genomes, as well as in complex microbial communities (metagenomes) from different environments [28].

### Potentially toxic metals and ecological systems

As we move towards a more sustainable future, our concern about long-term enrichment of PTM in agricultural soils is an arena for research and should be linked to the political agenda. Any ecologically sustainable future for our societies will depend upon intensive recycling of biological and non-biological materials. As PTM are found in so many products, a considerable part of this pollution will end up in our rivers, lakes, groundwater, and soils. Without taking these concerns into consideration, our efforts towards recycling and less use of new raw materials may have the potential for negative consequences linked to the toxicity of the metals. One of the more likely outcomes could be a link to an increasing problem of metal-resistance in bacteria that undermines our efforts to minimize the spread of AMR in bacteria from different niches.

Whereas ‘microbiota’ is defined as the microbial taxa within a given environment, the term ‘microbiome’ is defined as the genes and genomes of the microbiota, as well as the products of the microbiota and the host environment. Industrial inputs and the agronomic application of feed supplements, fertilizers, pesticides, and metal-contaminated sewage contribute to metal accumulation in the soil [29]. Toxic metals affect the growth, morphology, and metabolism of soil microorganisms, through functional disturbance, protein denaturation, and/or the destruction of the cell membrane (See Table 1). Soil microorganisms are essential for the decomposition of soil organic matter; any decrease in the microbial diversity or abundance may adversely affect nutrient absorption from the soil by plants [30].

Elevated levels of PTM in soils have significant impacts on the population size and overall activity of the microbial communities of soil in contaminated areas. Studies performed in Canada and China have described toxic metal contamination giving rise to shifts in microbial populations [31,32].

The presence of metal resistance genes in bacteria not only reflects the anthropocentric view of microbiology, with its embedded history of human antimicrobial use in infectious disease, but also reflects microbial exposure to the metals used in aquacultural and agricultural practices. Pre-dating all human uses, there is also the exposure of microorganisms to localized high levels of toxic metals from natural environmental releases over millennia [33].

However, most antimicrobial drugs are biologically produced and hence will be degraded relatively rapidly in most environments, with the exception of quinolones and tetracyclines, which are related to their chemical properties [34]. Thus, a future reduction in the use of antimicrobials as drugs for treating diseases in animals and humans, and as growth promoters in animal husbandry, may, over time, decrease the selective pressure from these substances. More research is needed to assist in our understanding of how increased levels of toxic metals influence, through co- and cross-selection, the complex global processes of resistance gene dynamics. It has been claimed that the long-term accumulation of toxic metals in agricultural soils has the potential to reduce soil productivity by inhibiting soil microbial and fauna populations, and may pose a risk to soil organisms, plants, animals, humans, and our ecosystems [18]. The bioavailability of metals generally decreases with increasing pH, organic matter content, and clay content of soil [4].

PTM or antimicrobial compounds can disperse through the environment via multiple and potentially complex pathways, and will remain in the environment unless physically removed, or through uptake by plants used for foods or eaten by animals. Transfer of PTM from crop soils into groundwater, rivers, and eventually marine waters depends on soil pH, cropping strategies, floods, other run-offs, etc. In practice we move from a relatively short time-scale for pharmaceutically produced antimicrobials, bacteria, and genes (months and years) to a more geological time-scale (decades, centuries) for metals. As long as biological and chemical fertilizers contain toxic metals, we must expect that levels in our agricultural soils will build up.

Information about the levels of PTM, like Zn and Cu, in soils in different countries is limited. There is also need for more data regarding the concentration of other PTM in soils, sewage, by-products, and fertilizers. Although present levels in agricultural soils may still be low, the long-term horizon of PTM in the environment indicates the importance of applying the precautionary principle and the understanding of the effects linked to increased efforts to recycle waste materials is in its infancy.

Thus, the levels of PTMs, such as Cu and Zn, added to animal feeds and used in fertilizer products must be discussed and rationalised in order to minimize environmental enrichment of these metals [6]. Furthermore, there is a need for more information regarding Cd, which is the predominant toxic metal in inorganic fertilizing products.

### Links between resistance towards potentially toxic metals and other antimicrobial agents

Thirty-nine articles fulfilled the criteria for evaluation and have been included in this article. Most publications are on sewage (n = 31) and most focus on the occurrence of toxic metal resistant bacteria, rather than on the ability of metals to induce resistance in bacteria in the environment. Eight articles were regarding PTM resistance in manure [35–42].

No studies were identified that addressed the potential release of toxic metal resistance genes to the environment, via fertilizing products. The majority of the studies included here are observational/descriptive studies that report on the co-existence of antimicrobial resistance determinants and toxic metal resistance determinants in bacteria. The bacterial species vary, and have been isolated from animals, humans, and environments in different countries and regions. Several variants of combinations of toxic metal resistance and antimicrobial resistance are described; a common feature is that the resistance-encoding genes are associated with mobile genetic elements. Some studies show direct genetic linkage, some show co-existence of resistance in the same isolate, some show co-existence in the same environment, but with unclear causal explanation.

In the articles evaluated, development of resistance against PTM was demonstrated in both sewage and manure. In several studies, several bacterial species were shown to have reduced susceptibility towards several potentially toxic metals, simultaneously. However, neither the source of heavy metal resistant bacteria nor the minimum co-selective concentration (MCC) for resistance induction is known. The Word Health Organization’s criteria used to categorize antimicrobials important to human health has been discussed elsewhere [43]. Co- or cross-resistance against highly important antimicrobials (streptomycin, tetracycline, neomycin) and critically important antimicrobials (e.g., amoxicillin, vancomycin, oxacillin, sulphonamides + trimethoprim, benzylpenicillin/phenoxymethylpenicillin, gentamicin) were identified in some of the bacterial isolates studied in these articles [35,38,39,41,44–50]. They suggest that there is a genetic linkage or direct genetic causality between genetic determinants to these widely divergent antimicrobials, and metal resistance. The data presented in these studies concur with the information presented in Table 2 regarding mechanisms of resistance against different PTM.

Based on the reviewed articles, we consider the probability for development and dissemination of PTM resistance in bacteria in sewage/manure and soil as illustrated in Table 3.

**Table 3.** Probability for development and dissemination of PTM resistance in bacteria in sewage/manure and soil.

Source of resistance	Comment
In organic fertilizing products	
Toxic metal resistant bacteria in fertilizing products	The probability of the simultaneous presence of AMR bacteria in sewage/manure is high (original articles reviewed).
Toxic metal resistance genes in fertilizing products and their mobility	The probability of the presence of toxic metal resistance genes in fertilizing products is high and transfer of such genes to bacteria in fertilizing products is possible.
Toxic metal residues and development of toxic metal resistant bacteria in fertilizing products	The probability of development of toxic metal resistance in susceptible bacteria due to toxic metals in fertilizing products is high.
In soil/environment	
Spread of toxic metal resistant bacteria from fertilizing products to soil/environment	The probability of spread of toxic metal resistant bacteria from sewage/manure to the soil/environment is high.
Spread of toxic metal resistance genes from fertilizing products to environmental bacteria	The presence and transfer of toxic metal resistance genes from fertilizing products to bacteria in soil is possible.
Development of toxic metal resistance in bacteria in soil/environment due to toxic metals in fertilizing products	Development of resistance in susceptible bacteria in soil due to the presence of toxic metals in fertilizing products is possible.

Although PTM like As, Hg, Co, Cd, Pb, and Ni may be present in the fertilizing products, the concentration may be low since they present as pollution. In contrast, trace metals like Cu and Zn are actively added to animal feed in many countries. In the following, we focus on two of the most critical PTM; Cu and Zn, because of their broad use as feed additive in animals in many countries and their high concentrations in manure used as fertilizers in agriculture.

#### Copper resistance

Several resistance mechanisms towards Cu have been described, including efflux pumps and cellular detoxification. These are both intrinsic and acquired characteristics of bacteria, and may occur in combination with AMR determinants in the same bacterial cells. One example of genetic linkage and co-occurrence on the same replicon is the pA17sv1 plasmid in enterococci that harbours resistance determinants towards Cu, macrolides, and glycopeptides [51]. The study [51] concluded, ‘Cu tolerance might contribute to the selection/maintenance of multi-drug resistant *Enterococcus* (including resistance to first-line antibiotics used to treat enterococcal infections) due to the use of Cu compounds (e.g. antiseptics/animal feed supplements)’. A proportion of the Cu-compounds used as antiseptics and animal feed supplements will eventually end up in organic fertilizers as manure and sewage, and exert induction of Cu tolerance and multidrug resistant enterococci in these environments due to positive selection of the plasmid host by Cu usage. However, the MCC for such induction is not known. Silveira, et al. [51] also found that enterococci containing Cu-resistance genes were more prevalent in samples from piggeries than from other animal production settings where Cu was used as feed supplement at lower concentrations than in piggeries, indicating positive selection for Cu-resistance determinants in the piggery setting.

In Denmark, glycopeptides were banned as growth promoters in animal production in 1995, and macrolides were banned in 1998. As the glycopeptide and macrolide resistance determinants (*vanA* and *erm*(B)) were shown to be located on the same plasmid in all Danish glycopeptide-resistant *E. faecium*, these bacteria did not decrease significantly until after 1998. Although the occurrence of the glycopeptide-resistant *E. faecium* decreased, they did not disappear completely [52]. Danish researchers have shown that a *tcrB* gene, which confers resistance to Cu in enterococci, is often located on the same transferable plasmid as the *vanA* and *erm*(B) determinants. Furthermore, the use of copper sulphate as a feed supplement for pigs has been shown to select for Cu resistance mediated by the *tcrB* gene in *E. faecium*, but the continued use of this feed supplement has not been able to maintain high levels of macrolide and glycopeptide resistance [53]. However, the selective pressure exerted by Cu-containing feed supplements may contribute towards maintaining low levels of these resistant bacteria in the gut microbiota. At re-exposure to glycopeptides or macrolides, the resistant bacteria may rapidly proliferate and become a dominant part of the enterococci population.

In Norway, the glycopeptide avoparcin was never approved for use in swine production, but was used as a feed additive in broiler and turkey production between 1986 and 1995, until implementation of a similar ban in Norway and Denmark. Several studies documented a continuing high prevalence of *vanA*-type vancomycin-resistant enterococci in the Norwegian poultry production several years after the ban [54–57]. The occurrence of vancomycin-resistant enterococci in poultry has been investigated as part of *the Usage of antimicrobial agents and occurrence of antimicrobial resistance in Norway (NORM-VET* programme: (https://www.vetinst.no/overvaking/antibiotikaresistens-norm-vet). In 2011, 16 % of broiler flocks were positive for *vanA*-type vancomycin-resistant *E. faecium*, and in 2013, 12 % of samples from turkeys were positive. All samples were analysed by a selective method for vancomycin-resistant enterococci [58,59]. These data show that there is a minor reservoir of vancomycin-resistant enterococci in Norwegian poultry production, but it is not known whether this reservoir is maintained due to the use of feed supplements containing copper sulphate.

The studies mentioned above are examples of genetic linkage to the same replicon, hence co-transferred between antimicrobial and toxic metals, and their respective microbial resistance determinants in the gut flora. This interplay is relevant for similar interactions that may occur in soil microbiota, as organic fertilizers containing toxic metals may have the same function as feed supplements in this context.

#### Zinc resistance

In Norway like many other countries, feed supplements containing Zn are approved for use in animals like pigs and poultry, and Zn is used for prevention of piglet diarrhoea. A report from 2014 concluded that Norwegian pigs are exposed to at least twice as much Zn as required to fulfil their physiological needs [60]. This excess Zn will end up in organic fertilizers when pig and poultry manure is used as such.

Jensen and co-workers concluded from a study in Denmark that ‘amendment of soils with pig slurry has led to a significant increase in soil concentrations of copper and zinc, especially in the latest monitoring period from 1998 to 2014’ [61]. Another published study from Denmark [62], demonstrated the ability of Zn, in addition to Cu, to co-select for AMR in bacteria in soil. In an experimental model, environmentally relevant levels of Cu and Zn co-selected for tetracycline resistance, while soil spiked with unrealistically high levels of tetracycline did not. The authors concluded that in some cases toxic metals may exert a stronger selection pressure for resistance to an antibiotic than the specific antibiotic itself. It has also been shown that Zn resistance of *S. aureus* of animal origin is strongly associated with resistance against methicillin, and it is suggested that the use of Zn in feed may have contributed to the emergence of livestock associated-MRSA in pigs [63].

In the natural water environment (water and sediments), Cd, Cu, Ni, Hg, Co, Pb, and Zn concentrations frequently reach levels that exceed their respective MCC values for several bacterial species and, therefore, may drive co-selection. Although several studies have investigated co-selection in the aquatic environment, only a few publications consider soil environments; reviewed by Seiler and Berendonk [5]. In soil, Cu levels reach concentrations that are reported as potentially co-selective for antibiotic resistance genes [3]. In contrast, a Zn MCC for soil samples could not be determined because Knapp et al. [3] did not detect an increasing abundance of antibiotic resistance genes in correlation with elevated Zn concentrations. However, the Zn concentrations of soil samples investigated by Knapp et al. [3], were relatively low compared with those reported from other soils and maybe within the no-effect range.

Although these studies are concrete examples of apparent genetic linkage (e.g. co-selection) of antimicrobial and metal resistance, systematic data on high-risk hot spots (such as from Cu and Zn in manure) for human-animal-environmental bacterial interactions, are scarce.

No studies were identified that investigated the abundance of AMR genes (resistome) in the different environmental samples. Therefore, in general we cannot distinguish between the natural resistome and elevated abundance of antibiotic resistance genes in environmental samples; detecting an increase of antibiotic resistance genes in environmental samples is not easy.

## Discussion

### PTM in soil and fertilizing products

There is no systematic monitoring of toxic metals in soils neither in Norway nor from other countries. Based on available data, it appears that the levels are highly variable, depending on geology and characteristics of the soil. Human activities over centuries, such as industrial, urban, agricultural, and aquacultural activities, have resulted in increased levels of toxic metals in the environment, especially in areas where such activities have been intensive or combined. It is currently not possible to relate these partly unknown and variable levels to their impact on development, dissemination, and persistence of AMR.

As for soil, data on toxic metals in fertilizing materials are fragmented and limited. Fertilizing materials, in the form of sewage sludge or livestock manure, will either add to or ‘dilute’ already existing levels of these toxic metals in soil, especially in areas of intensive agriculture. The additive effect of toxic metals in fertilizing materials must be regarded from a long-term perspective, as these metals accumulate in the environment. It is not possible to relate these partly unknown and variable levels with their impact on development, dissemination, and persistence of AMR. However, areas of intensive agriculture may be regarded as ‘hot-spots’ for interactions between bacteria of environmental, animal, and human origin, and the toxic metals. In this review paper, we focus in particular on the metals that are actively added to the environmental cycles through animal feed (Zn, Cu). Although other PTM may also be relevant, we consider the levels of these to be low. In sewage sludge, Cd is considered the most important toxic metal with respect to environmental contamination. As Zn and Cu are added to animals like swine and poultry feed in levels exceeding these animals’ physiological needs, these metals are the most relevant to consider in livestock manure.

### Development, spread and persistence of AMR

Development of AMR can be partly regarded as a dose- and time dependent response to exposure to different drivers for resistance. There is a strong indication that the PTM evaluated in this review article are driving forces for the development of AMR in exposed bacteria, but the dose- and time exposures most likely to have greatest impact are not known. The naturally occurring background resistance in environmental bacteria complicates our estimation of the effect of toxic metal exposure on the development of AMR, and we are currently unable to distinguish readily between the natural resistome and an elevated abundance of AMR in different environmental samples due to metal contamination. Heavy metal driven co-selection of AMR in environments impacted by agriculture and aquaculture should especially focus on Cd, Cu and Zn as co-selecting factors for the development of AMR.

The persistence and dissemination of AMR can occur when metal resistance within bacteria confers cross- and/or co-resistance to particular antibiotics. The term minimum co-selective concentration (MCC) of metals was recently introduced as a term that specifies the minimum toxic metal concentration that correlates with detection of increased AMR. The use of the MCC term is an interesting approach, and, in the future, may prove to be a useful method for assessment of the co-selective effect of a PTM. However, considerable field- and laboratory-based research is needed to establish the MCC-approach as an acknowledged and unifying method comparable to the MIC methods used by EUCAST and CLSI.

Many examples of cross- and co-resistance between toxic metals and antibiotic resistance are described in the literature. Most important are those cases where toxic metal resistance determinants are genetically linked to resistance determinants towards *highly important* and *critically important* antibiotics. Emergence of livestock-associated methicillin-resistant *S. aureus* in pigs is one of the most alarming examples of AMR. The association between resistance to Zn and methicillin-resistance in *S. aureus* of animal origin suggests that the use of Zn as a feed supplement could have contributed to contribute to the persistence, amplification, and dissemination of MRSA in pigs, rather than initial development.

Traits conferring resistance to antimicrobial compounds have been present in some bacteria since times pre-dating human society, probably as defence mechanisms to antibiotics produced by bacterial communities. The various mechanisms for current resistance are most often known and understood within an evolutionary perspective. However, we do not fully understand the mechanisms behind the persistence of AMR. Removal of selective conditions for development and spread of resistance may result in the levels of resistance decreasing, but not necessarily lead to a full disappearance. The presence of a minor proportion of a bacterial population that is resistant means that it has the potential to outcompete the remaining population should that population again be exposed to a corresponding antibiotic or toxic metal. The interaction between antibiotics/PTM/disinfectant agents and bacteria may be a major cause for development of AMR in bacteria.

Through the use of fertilizing materials, the bacterial influx to the environment belongs to the large group of gut microbiota. These bacteria are adapted to the intestinal environment, and their environmental survival abilities are variable. Much of this microbiota will die out and not influence the environmental microbiota over time. Composting of livestock manure and the production process of sewage sludge reduces the number of microbes added to the soil and environment. However, data on the long-term fate of ARB and AMR genes originating from an intestinal environment are fragmented and limited.

### Uncertainties and lack of knowledge

A number of uncertainties have been identified related to the probability of formation of, and dissemination of, AMR due to the release of toxic metals to the environment. Many of these uncertainties are due to our limited understanding of the complex processes occurring at spatiotemporal scales not fully amendable to experimental investigation. A quantitative framework remains to be developed. There are many data gaps and detailed data on the current and future use of toxic metals, along with their environmental levels, are not readily available. Without these data, estimating the selective levels and MCC that could potentially induce increased AMR is challenging. The present methods for determining AMR in environmental samples are primarily culture-based laboratory studies (± antimicrobials) or using culture-independent methods and examining for the presence of antimicrobial resistance genes (ARGs), using PCR or sequencing. The latter methods do not fully capture the potential for co-selection with toxic metals. There are also uncertainties regarding the ability of toxic metal-resistant bacterial strains to colonize humans or animals, the extent of such colonization, and the ability of their resistance genes to be transferred to resident bacterial species in the environment.

There is lack of knowledge regarding links between the level and concentration of PTM in fertilizing products and soil and development of resistance in bacteria. Data regarding the routes and frequencies of transmission of AMR from bacteria of environmental origin to bacteria of animal and human origin were lacking in the published articles reviewed here. Due to the lack of such data, it is difficult to estimate the probability of development, transmission, and persistence of PTM resistance in the Norwegian environment. More research is needed to explain the relationship between development of resistance against potential toxic metals and resistance toward antimicrobial agents in bacteria.
